# Treatment persistence, lipid lowering, and 3-year clinical outcomes in patients at very high cardiovascular risk on PCSK9 monoclonal antibodies

**DOI:** 10.1007/s00392-025-02719-z

**Published:** 2025-08-04

**Authors:** Klaus G. Parhofer, David Pittrow, Andreas L. Birkenfeld, Uwe Fraass, Bernd Hohenstein, Carsten Siegert, Jens Klotsche, Elisabeth Steinhagen-Thiessen, Stefan Dexl, Volker J. J. Schettler, Ulrich Laufs

**Affiliations:** 1 Medizinische Klinik und Poliklinik IV, Ludwig-Maximilians-Universität, München, Deutschland; 2 Institut für Klinische Pharmakologie, Medizinische Fakulät, Technische Universität, Dresden, Deutschland; 3 Innere Medizin IV - Diabetologie, Endokrinologie und Nephrologie am Universitätsklinikum, Tübingen, Deutschland; 4 Amgen GmbH, München, Deutschland; 5 Nephrologisches Zentrum, Villingen-Schwenningen, Deutschland; 6 Epidemiologie, Deutsches Rheuma-Forschungszentrum, Berlin, Deutschland; 7 Medizinische Klinik für Endokrinologie und Stoffwechselmedizin, Charité - Universitätsmedizin Berlin, Berlin, Germany; 8 Nephrologisches Zentrum, Göttingen, Germany; 9 Klinik und Poliklinik Für Kardiologie, Universitätsklinikum Leipzig, Leipzig, Deutschland; 10 Innovationszentrum Real World Evidence, GWT-TUD GmbH, Dresden, Deutschland

**Keywords:** Real word evidence, High risk, LDL cholesterol, Persistence, Secondary prevention

## Abstract

In a cohort of patients with dyslipidemia at very high cardiovascular risk, we investigated differences in LDL-C lipid target achievement, clinical outcomes, and persistence rates between users and non-users of PCSK9 monoclonal antibodies (PCSK9-mAb) over a 3-year observation period. The prospective, multi-center observational study included 1695 patients with dyslipidemia. Eligible patients were adults with familial or non-familial hypercholesterolemia, mixed dyslipidemia, or other therapy-refractory lipid disorders in line with the G-BA reimbursement regulations. Treatment decisions, including PCSK9-mAb administration, were made at the discretion of the treating physician. At baseline, 804 (47.4%) patients received PCSK9-mAb therapy, and 891 (52.5%) did not. There were 42 (4.7%) new PCSK9-mAb receivers during the follow-up. Median propensity-score adjusted LDL-C levels in PCSK9-mAb non-receivers decreased over time from 106.0 to 68.4 mg/dL. LDL-C in PCSK9-mAb receivers dropped from 112.5 mg/dL at baseline to 58.0 mg/dL at 3 years, consistently outperforming non-receivers. Target LDL-C goal attainment (< 55mg/dL) after 3 years was higher in the PCSK9-mAb group (43.2% vs. 34.5%). Persistence with PCSK9-mAb therapy over 3 years since treatment initiation was high (91.5%). Higher discontinuation rates of PCSK9-mAb were associated with baseline statin intolerance (HR = 2.3, *p* = 0.012). The use of PCSK9-mAb was associated with numerically fewer cardiovascular events (9.3 versus 15.7 per 100 patient-years, *p* not significant) and lower hospitalization rates due to cardiovascular events compared to non-users (6.3 versus 12.4 per 100 patient years, *p* = 0.001). This study underscores the real-world efficacy and safety of PCSK9-mAb therapy in achieving sustained LDL-C reduction. Identifier: Clinicaltrials.gov NCT03110432.

**Graphical Abstract:**

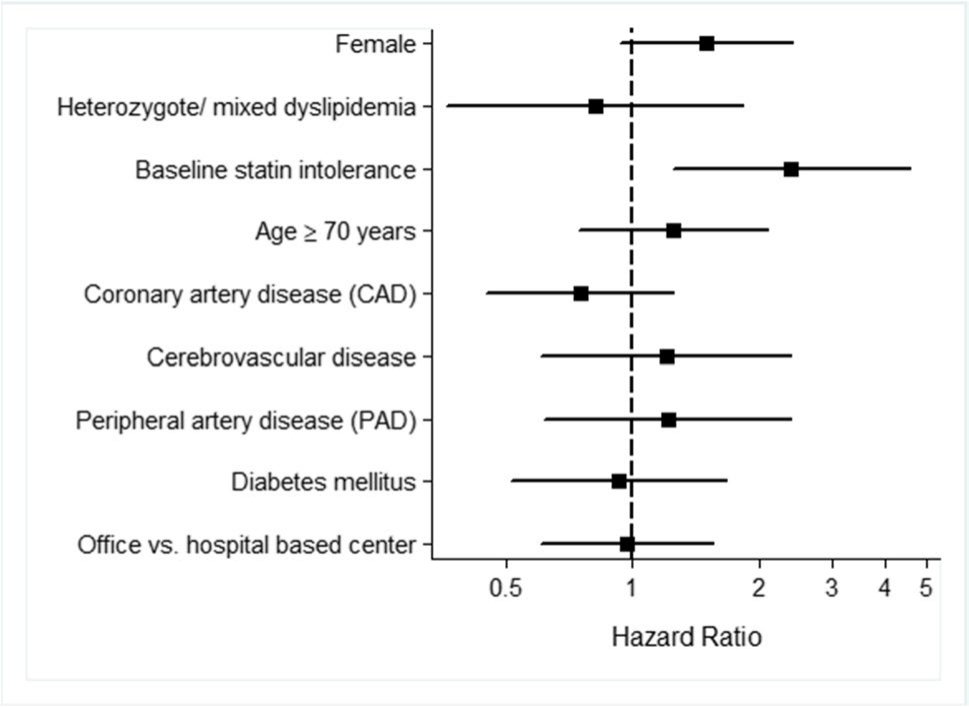

**Supplementary Information:**

The online version contains supplementary material available at 10.1007/s00392-025-02719-z.

## Background

Consistent evidence highlights the central role of lowering low-density lipoprotein cholesterol (LDL-C) in preventing cardiovascular (CV) events. The EAS/ESC dyslipidemia guidelines of 2016 recommended a target of < 70 mg/dL (1.8 mmol/L) or a 50% reduction in treatment naïve LDL-C levels [1]. Based on data from several randomized trials, these guidelines were updated in 2019 to advocate for lower targets, recommending LDL-C levels of < 55 mg/dL (1.4 mmol/L) and a significant reduction exceeding 50% for patients at very high risk [2].

In addition to traditional interventions such as lifestyle modification, lipid-lowering therapy based on statins, and ezetimibe, the 2019 guidelines introduced proprotein convertase subtilisin kexin type 9 (PCSK9) inhibitors into the treatment algorithm for patients with very high CV risk and elevated LDL-C levels. Fully human PCSK9 monoclonal antibodies (PCSK9-mAb), approved in 2015, including evolocumab and alirocumab, have demonstrated the ability to further reduce LDL-C levels by 50–60% on top of existing lipid-lowering therapies, with a favorable safety profile [3]. Large-scale outcome trials have confirmed that these antibodies significantly reduce CV risk in patients with atherosclerotic CV disease (ASCVD) [4, 5].

Despite their efficacy, access to PCSK9-mAb has been restricted in various countries due to regulatory and reimbursement constraints [6]. In Germany, for example, the Federal Joint Committee (G-BA) has limited PCSK9-mAb reimbursement to a specific subset of ASCVD patients who do not achieve LDL-C targets despite optimal oral lipid-lowering therapies [7, 8]. The gap between the number of patients eligible for PCSK9-mAb treatment and those who actually receive it has resulted in a need to understand the factors that influence the prescription patterns for PCSK9-mAb treatment.

The primary objective of the PERI-DYS study was to compare the target goal attainment in LDL-C values and general differences in patient characteristics in two cohorts of dyslipidemia patients at very high CV risk: those receiving PCSK9-mAb and those eligible but not receiving these inhibitors. In addition, we were interested in gaining further insights into relevant clinical outcome parameters. Previous cross-sectional analyses indicated that PCSK9-mAb receivers had higher baseline LDL-C levels and a higher prevalence of statin intolerance compared to non-receivers [9].

The present analysis focusses on differences in LDL-C target achievement, persistence rates, and clinical outcomes, between PCSK9-mAb receivers and non-receivers over the full 3-year observation period.

## Methods

### Study design and setting

The PERI-DYS study is a prospective, multi-center, observational registry with a 3-year follow-up period. Eligible participants included physicians from hospital and office settings, primarily those experienced in managing dyslipidemia. These physicians encompassed internists specialized in cardiology, nephrology, diabetology, and endocrinology (including lipidology), with cardiologists comprising the largest group of investigators.

### Ethical approval and informed consent

Before the initiation of the study, the research protocol and the informed consent form were approved by the institutional review board at the Medical Faculty of the Technical University of Dresden, Germany (reference number: EK 4761120166). Written informed consent was obtained from all participating patients. The study was registered with the Paul Ehrlich Institute under NIS384 and on ClinicalTrials.gov with the identifier NCT03110432.

The first patient was enrolled on 16 May 2017, and the last patient entered the study on 30 June 2021; the final documented visit took place on 17 April 2024. Recruitment thus began approximately 18 months after the initial market launch of PCSK9-mAb in 2015 and the implementation of corresponding G-BA reimbursement regulations in Germany. It concluded in mid-2021, at which point the updated ESC/EAS dyslipidemia guidelines had already been in effect for nearly 2 years.

### Study cohort and data collection

The inclusion criteria for patients were as follows:

1. Homozygous familial hypercholesterolemia: Patients for whom pharmaceutical and dietary interventions had proven insufficient.
2. Heterozygous familial hypercholesterolemia under consideration of the total familial risk (diagnosed clinically; DLCN score not mandatory).
3. Heterozygous familial or non-familial hypercholesterolemia, or mixed dyslipidemia: therapy refractory course

- maximal dietary and pharmaceutical lipid lowering therapy—in any case documented over a 12-month period
- unsatisfactorily lowered LDL-C value (and thus with an indication for LDL apheresis)
- confirmed vascular disease
- other risk factors for cardiovascular events

“Very high cardiovascular risk” was defined by the presence of established ASCVD and/or multiple major risk factors per ESC/EAS 2016/2019 criteria.

In addition to these medical criteria, patients were required.

- to be at least 18 years old and
- to provide written informed consent
- to be suitable for inclusion in a long-term study addressing adherence, comorbidities, and prognosis.

The sole exclusion criterion was concurrent participation in a randomized clinical trial.

Participating sites were instructed to enroll a comparable number of patients receiving PCSK9 inhibitor (PCSK9-mAb) treatment and those without PCSK9-mAb therapy. This approach was designed to prevent overrepresentation of the former group and provide a broader perspective on the treatment landscape.

Data collection focused on capturing detailed clinical and therapeutic histories to ensure comprehensive assessment and adherence to the study criteria.

### Treatment and data documentation

In this study, all diagnostic and therapeutic decisions were made at the discretion of the treating physician, provided that only commercially available drugs were used. The administration of PCSK9-mAb therapy was required to follow the recommended posology specified in the respective package insert. Additionally, treatment with either of the two registered PCSK9-mAb antibodies had to adhere to the criteria outlined in the final versions of the “prescription restriction” documents for Repatha® or Praluent®.

Patients were permitted to switch between PCSK9-mAb brands (e.g., from evolocumab to alirocumab, or vice versa) or to/from other lipid-lowering therapies.

Data collection included information on concomitant diseases, which was recorded using both checkboxes for predefined conditions and free-text fields for additional details. Information on lipid-lowering therapies (LLT) and cardiovascular medications was gathered and coded using WHO-DD Drugs Insights with ATC codes. For LLT, the name, dose, and exact treatment dates were documented, including reasons for discontinuation where applicable.

Treatment discontinuation and the reasons for discontinuation were reported by the treating physician. For the calculation of persistence to PCSK9-mAb treatment, the reported actual treatment initiation date was used instead of the study start date. This approach ensures that the calculation reflects the actual exposure to therapy and is not biased by delays between treatment initiation and study inclusion.

Clinical events were systematically recorded on a dedicated case report form (CRF) to ensure accurate and consistent data collection. Deaths were categorized based on cause, distinguishing between cardiovascular (CV) deaths, non-cardiovascular deaths, and those of unknown origin. Acute coronary syndrome (ACS) events were further classified into ST-elevation myocardial infarction (STEMI), non-ST-elevation acute coronary syndrome (NSTE-ACS), angina pectoris, and cases with an unknown classification. Cerebrovascular events were categorized by ischemic stroke, hemorrhagic stroke, transient ischemic attack (TIA), other cerebrovascular conditions, and events of unknown type. Hospitalizations were recorded for CV events, side effects, other causes, or an unknown reason. Rehabilitation stays distinguished between those related to cardiovascular conditions and those due to other health issues.

Quality of life was assessed using the EuroQoL visual analogue scale (VAS), which ranges from 0 (worst imaginable health state) to 100 (best imaginable health state). Patients were asked to rate their current overall health status at each study visit.

Statin intolerance was physician-reported, based on patient history of adverse effects. Where possible, muscle symptoms were documented separately.

Data recording was conducted using a web-based electronic case report form (CRF), completed by designated personnel at the participating sites. To ensure data quality and accuracy, statistical measures were applied for verification, and on-site monitoring was performed as part of the quality assurance process.

The statistical analysis used the final dataset after database lock. Categorical variables were summarized in frequency tables, with absolute and relative frequencies reported. Continuous variables were analyzed using descriptive statistics, including the sample median and interquartile range. Additionally, the mean and standard deviation were reported where appropriate. Given the distribution patterns of lipid parameters, this paper presents medians of calculated LDL-C values and other lipid parameters to provide a more accurate representation of the data.

To evaluate the LDL-C course, in this analysis, a propensity score (PS) was estimated to balance clinical characteristics between the two treatment groups using logistic regression [10]. The PS included baseline demographic and clinical characteristics, including comorbidities and additional treatments, to account for potential confounding factors. An inverse probability weight was calculated for the two treatment groups: for patients receiving PCSK9-mAb therapy, at baseline (pre-treated and newly treated), the weight was defined as 1/PS, and for those not receiving PCSK9-mAb therapy, the weight was 1/(1-PS). We refer to the supplemental material for more details and validation of the propensity score.

Major adverse cardiac and cerebrovascular events (MACCE) were defined as a composite outcome, including death from cardiac cause, acute coronary syndrome, and cerebrovascular events. The incidence of clinical events was reported as the number of events per 100 patient-years, based on the allocation of patients to PCSK9-mAb treatment at baseline. A “per protocol” analysis was conducted as a sensitivity analysis. Clinical events were reassigned based on their temporal relationship to treatment: events occurring in patients after the discontinuation of PCSK9-mAb therapy were counted in the non-receiver group, while events in non-receivers who later initiated PCSK9-mAb therapy were reassigned to the receiver group. The association between sociodemographic factors and comorbidities with the onset of clinical events was determined by Cox proportional hazard analysis.

Statistical analyses were conducted using the software package SAS version 9.3 (SAS Institute Inc, Cary, NC, USA), or a higher version if available.

## Results

### Patient disposition

A total of 1695 patients who met all eligibility criteria were included in the final analysis. The median follow-up duration was 35.6 months (approximately 3.0 years), with a median of 6.0 visits per patient. Forty patients (2.4%) had a baseline visit but did not attend any follow-up visits. The majority of patients, 1405 (82.9%), completed the 36-month follow-up, and 982 patients (57.9%) attended all six planned visits.

**Fig. 1:**
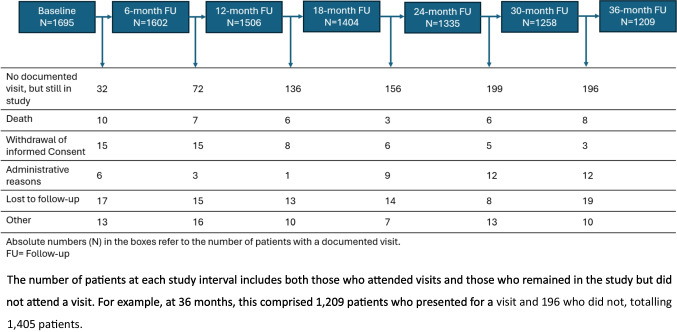
Patient flow over time

Study discontinuation occurred in 276 cases (16.3%). Reasons for discontinuation included withdrawal of consent (52 cases, 3.1%), administrative reasons (43 cases, 2.5%), loss to follow-up (86 cases, 5.1%), death (40 cases, 2.4%), and other reasons (69 cases, 4.1%). A detailed overview of the patient flow is provided in Fig. 1.

### Patient characteristics and comorbidities

Patient characteristics are summarized in Table 1. The majority of participants (*n* = 1,595; 94.1%) were diagnosed with heterozygous familial or non-familial hypercholesterolemia or mixed dyslipidemia, while 95 (5.6%) had confirmed familial heterozygous hypercholesterolemia, and 5 (0.3%) had familial, homozygous hypercholesterolemia. Statin intolerance was reported in 711 patients (41.9%). Among the leading comorbidities, arterial hypertension was the most prevalent, followed by diabetes mellitus and heart failure. Baseline lipid values are shown in Table 2.

**Table 1:** Characteristics, comorbidities at baseline

Parameter	PCSK9-mAb receiver	PCSK9-mAb non-receiver	*p* value
	(*n*=804)	(*n*=891)	
Homozygous familial hypercholesterolemia	4 (0.5%)	1 (0.1%)	0.056 *
Heterozygous familial hypercholesterolemia	54 (6.7%)	41 (4.6%)	
Non-familial/mixed dyslipidemia	746 (92.8%)	849 (95.3%)	
Sex, female	306 (38.1%)	281 (31.5%)	0.03
Age (years, mean, SD)	62.3 (10.3)	64.3 (12.3)	0.02
BMI, kg/m^2^	28.4 (4.7)	28.2 (4.7)	0.26
Statin intolerance	551 (68.5%)	160 (17.9%)	<0.001
Adverse effects of statins in patient history			<0.001
Myalgia	464 (57.7%)	128 (14.4%)	
Myositis	6 (0.7%)	7 (0.8%)	
Rhabdomyolysis	0 (0.0%)	2 (0.2%)	
**Comorbidities, %**			
Arterial hypertension	637 (80.0%)	748 (84.7%)	0.02
Diabetes mellitus	194 (24.4%)	256 (28.7%)	0.02
CAD	594 (74.6%)	608 (68.9%)	0.01
CVD	108 (13.6%)	96 (10.9%)	0.24
PAD	102 (13.0%)	198 (22.9%)	<0.001
Any combination CAD, CVD, PAD	659 (82.0%)	721 (80.9%)	0.63
Chronic kidney disease	86 (10.8%)	104 (12.0%)	0.03

*BMI *body mass index, *CAD *coronary artery disease, *CVD* cardiovascular disease, *PAD* peripheral artery disease, *SD* standard deviation

* Chi2 test over 3 categories

**Table 2:** Lipid values at baseline

Lipid values	PCSK9-mAb receiver (*n* = 804)	PCSK9-mAb non-receiver (*n* = 891)	*p* value†
	Median (IQR)	Median (IQR)	
Total cholesterol	156.2 (122.0–201.0)	171.5 (140.0–215.4)	< 0.001
LDL-C	81.2 (52.6–131.0)	99.0 (74–138.4)	< 0.001
HDL-C	50.3 (42.0–62.0)	47.2 (40.0–57.0)	< 0.001
Non-HDL-C	89.0 (56.0–137.7)	116.0 (81.0–165.0)	< 0.001
Triglycerides	139.9 (94.0–208.0)	135.0 (95.6–199.0)	0.529
Lp(a)	45.9 (7.0–93.0)	37.0 (10.2–104.0)	0.712

Values are medians (interquartile ranges). Note that the PCSK9-mAb receiver group includes both patients who were already on PCSK9-mAb therapy at enrollment (i.e., treatment effects already present) and those newly prescribed PCSK9-mAb (i.e., treatment effects expected to emerge over the following weeks)

† *p* value for comparison of the two groups from Wilcoxon rank-sum test

### Lipid lowering treatment

At baseline, 804 (47.4%) were receiving PCSK9-mAb therapy, while 891 (52.6%) were not. During the follow-up period, PCSK9-mAb therapy was initiated in 42 patients (5.0%) from the non-receiver group, categorizing them as new users. Table 3 illustrates the rates of treatment with PCSK9-mAb, statins, ezetimibe, and inclisiran over time.

**Table 3:** Treatment over time

	PCSK9-mAb receiver (*n* = 804)	PCSK9-mAb non-receiver (*n* = 891)
**Treatment, %**		
On PCSK9-mAb at baseline (BL)	100.0	0.0
At 1 year	96.3	10.4
At 2 years	94.4	12.2
At 3 years	93.6	11.4
On statins at BL	53.9	91.7
At 1 year	57.0	90.9
At 2 years	58.4	90.8
At 3 years	57.0	92.8
On ezetimibe at BL	49.0	41.1
At 1 year	48.9	48.7
At 2 years	47.9	52.0
At 3 years	47.2	51.2
On inclisiran at BL	0.2	0.0
At 1 year	0.8	0.3
At 2 years	1.5	0.4
At 3 years	2.8	0.8

At baseline, 53.9% of PCSK9-mAb receivers were also on statin therapy, with rates ranging from 57.0 to 58.4% in subsequent years. In contrast, 91.7% of PCSK9-mAb non-receivers were on also statins at baseline (7.2% on regular, 38.7% on moderate, and 54.7% on high-intensity statins), with rates remaining consistently high between 90.8 and 92.8% throughout the follow-up.

Regarding ezetimibe use, 49.0% of PCSK9-mAb receivers were on ezetimibe at baseline, with rates ranging from 47.2 to 48.9% over time. Among PCSK9-mAb non-receivers, 41.1% were on ezetimibe at baseline, with usage increasing to 48.7–52.0% during follow-up. Other lipid-lowering therapies were prescribed in less than 9% at any time point.

The RNAi therapeutic inclisiran was prescribed in 17 cases at 3 years (2.8%) in PCSK9-Mb receivers and in 5 cases at 3 years (0.8%) in PCSK9-mAb non-receivers.

### Factors associated with PCSK9-mAb discontinuation

Among the 804 patients receiving PCSK9-mAb therapy, the overall discontinuation rate was 8.5% over a median treatment duration of 36 months. The most commonly reported reasons for discontinuation were adverse drug reactions (2.7%), followed by patient withdrawal (1.7%), lack of efficacy as stated by the treating physician (0.6%), LDL-C target achievement (0.1%), administrative reasons (0.6%), and other unspecified causes (2.6%).

**Fig. 2:**
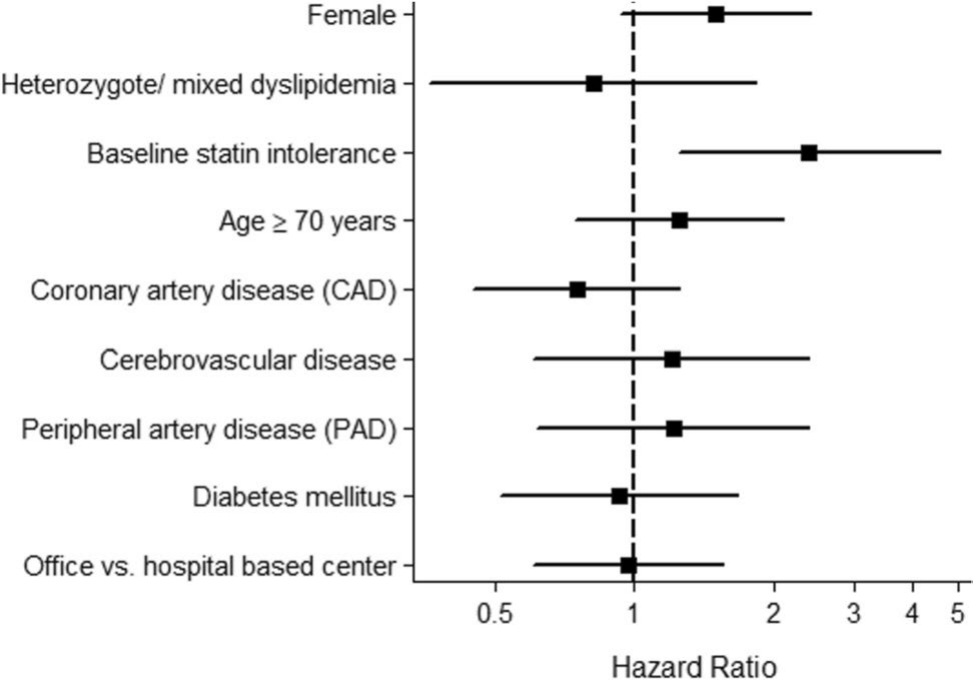
Factors associated with discontinuation of PCSK9-mAb therapy

Discontinuing PCSK9-mAb therapy was significantly associated with baseline statin intolerance with a hazard ratio (HR) of 2.3 (95% CI 1.2–4.4, *p* = 0.012). Other parameters investigated were female sex (HR 1.5, 95% CI 0.9–2.4, *p* = 0.107), age ≥ 70 years (HR 1.3, 95% CI 0.8–2.2, *p* = 0.297), heterozygous or mixed dyslipidemia (HR 0.9, *p* = 0.732), coronary artery disease (HR 0.7, *p* = 0.255), cerebrovascular disease (HR 1.1, *p* = 0.839), peripheral artery disease (HR 1.2, *p* = 0.671),) office vs. hospital based initiation of therapy (HR 0.97, *p* = 0.904), and diabetes mellitus (HR 1.0, *p* = 0.878), but these were not statistically significantly associated with discontinuation of PCSK9-mAB (Fig. 2).

Factors associated with discontinuation of PCSK9-mAb therapy, presented as hazard ratios (HR) with 95% confidence intervals. Baseline statin intolerance was significantly associated with increased risk of discontinuation (HR 2.2, *p* = 0.012).

Other variables, including sex, age ≥ 70 years, and comorbid conditions such as coronary artery disease, diabetes mellitus, and peripheral artery disease, and type of care setting did not reach statistical significance. The dashed line indicates the reference value (HR = 1.0).

### LDL-C cholesterol reduction unadjusted and after propensity score adjustment

**Fig. 3:**
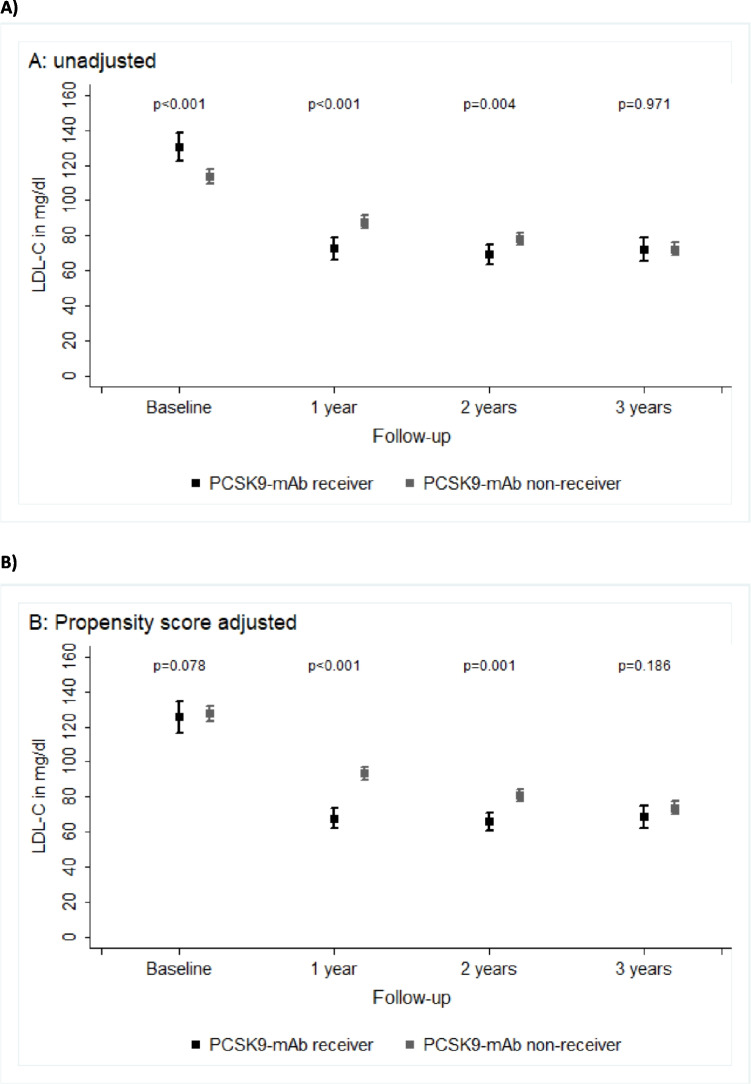
LDL-C values over time **A** unadjusted and **B** adjusted for propensity score

Patients who received PCSK9-mAb newly at inclusion (266 patients with values at baseline) had an (unadjusted) median LDL-C at baseline of 120.3 mg/dL, at 6 months 65.2 mg/dL, at 1 year 62.0 mg/dL, at 2 years 60.0 mg/dL, and at 3 years 63.4 mg/dL. PCSK9-mAb non-receivers (*n* = 804 with values at baseline) had an LDL-C at baseline of 99.0 mg/dL, at 6 months 83.0 mg/dL, at 1 year 79.0 mg/dL, at 2 years 72.0 mg/dL, and at 3 years 65.4 mg/dL (Fig. 3A).

After propensity score adjustment in patients with a 3-year follow-up and documented LDL-C values, LDL-C values in newly treated PCSK9-mAb receivers (*n* = 442 with baseline values) showed a significant reduction, with a median LDL-C of 112.5 mg/dL at baseline, 56.0 mg/dL at 1 year, 52.0 mg/dL at 2 years, and 58.0 mg/dL at 3 years. In comparison, PCSK9-mAb non-receivers (*n* = 420 with baseline values) showed median LDL-C levels of 106.0 mg/dL at baseline, 85.0 mg/dL at 1 year, 78.0 mg/dL at 2 years, and 68.4 mg/dL at 3 years (Fig. 3B). Overall, 39.6% of the total cohort achieved their LDL-C target of < 55mg/dL. The target attainment rate was higher in the PCSK9-mAb group (43.2% vs 34.5%).

### Clinical events

**Fig. 4:**
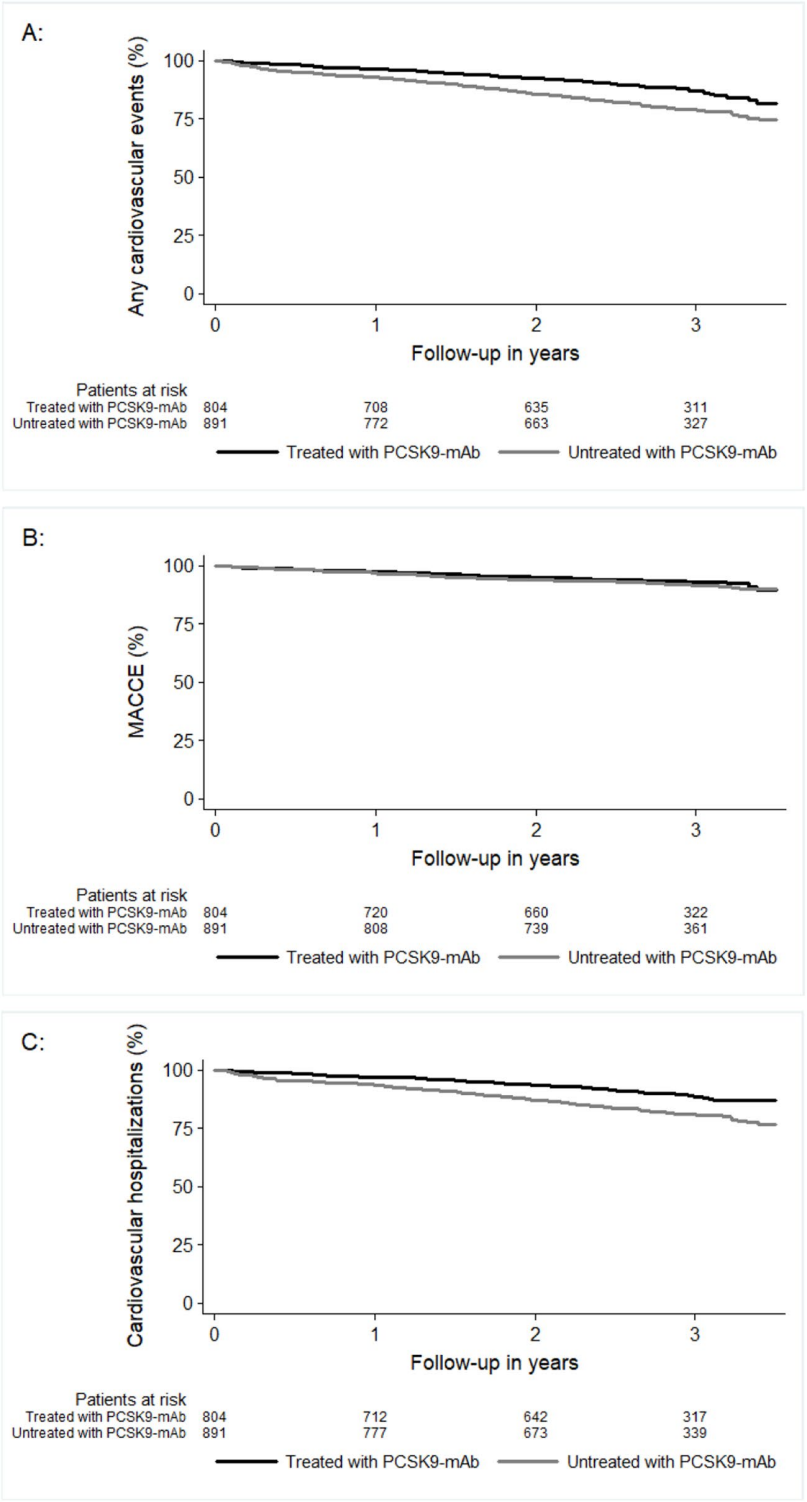
Kaplan Meier curves of **A** any cardiovascular events, **B** severe cardiac and cerebrovascular complication (MACCE) and **C** cardiovascular hospitalizations. Time to first event is shown by Kaplan Meier curves

An overview of clinical events, both overall and by specific event type, is provided in Table 4 and Fig. 4 A–C. Cardiovascular events occurred numerically less frequently in PCSK9-mAb receivers compared to non-receivers, with rates of 9.3 versus 15.7 events per 100 patient-years (not significant). Similarly, severe cardiac and cerebrovascular complications (MACCE) were numerically slightly less frequent in PCSK9-mAb receivers (3.1 versus 3.7 events per 100 patient-years, not significant), as were hospitalizations due to CV events (6.3 versus 12.4 events per 100 patient-years, *p* = 0.001).

**Table 4:** Clinical events in total and rate by 100 patient years

Event	PCSK9-mAb receiver		PCSK9-mAb non-receiver		PCSK9-mAb receiver versus PCSK9-mAb non-receiver
	Events (rate per 100 years)	Patients with event	Events (rate per 100 years)	Patients with event	RR (95% CI), *p*-value
**Any event**	**314 (14.7)**	163 (20.3%)	**565 (23.7)**	251 (28.2%)	0.88 (0.75; 1.03), 0.123
**Any cardiovascular event**	**199 (9.3)**	97 (12.1%)	**375 (15.7)**	174 (19.5%)	0.86 (0.71; 1.04), 0.123
**Severe cardiac and cerebrovascular complication (MACCE)**	**56 (2.6)**	42 (5.2%)	**67 (2.8)**	54 (6.1%)	0.96 (0.70; 1.32), 0.795
**Death**	**14 (0.7)**	14 (1.7%)	**29 (1.2)**	29 (3.3%)	1.33 (0.89; 1.97), 0.159
Death from cardiovascular cause	3 (0.1)	3 (0.4%)	7 (0.3)	7 (0.8%)	0.95 (0.41; 2.16), 0.895
Death from non-cardiovascular cause	3 (0.1)	3 (0.4%)	8 (0.3)	8 (0.9%)	1.03 (0.46; 2.31), 0.935
Death from unknown cause	8 (0.4)	8 (1.0%)	14 (0.6)	14 (1.6%)	1.53 (0.91; 2.57), 0.107
**Acute coronary syndrome**	**45 (2.1)**	41 (5.1%)	**55 (2.3)**	45 (5.1%)	0.85 (0.62; 1.16), 0.308
Acute coronary syndrome STEMI	3 (0.1)	3 (0.4%)	13 (0.5)	12 (1.4%)	1.32 (0.74; 2.34), 0.342
Acute coronary syndrome NSTE-ACS	7 (0.3)	7 (0.9%)	12 (0.5)	10 (1.1%)	0.69 (0.50; 0.95), 0.023
Acute coronary syndrome angina pectoris	32 (1.5)	30 (3.7%)	24 (1.0)	23 (2.6%)	1.10 (0.96; 1.25), 0.184
Acute coronary syndrome unknown	2 (0.1)	2 (0.3%)	5 (0.2)	4 (0.5%)	0.75 (0.46; 1.22), 0.245
**Cerebrovascular event**	**15 (0.7)**	10 (1.2%)	**16 (0.7)**	11 (1.2%)	1.09 (0.79; 1.49), 0.606
Cerebrovascular event ischemic stroke	4 (0.2)	4 (0.5%)	2 (0.1)	2 (0.2%)	0.99 (0.93; 1.06), 0.759
Cerebrovascular event hemorrhagic stroke	0 (0.0)	0 (0.0%)	0 (0.0)	0 (0.0%)	-
Cerebrovascular event TIA	1 (0.0)	1 (0.1%)	0 (0.0)	0 (0.0%)	-
Cerebrovascular event unknown cause	1 (0.1)	2 (0.3%)	0 (0.0)	0 (0.0%)	-
Cerebrovascular event other cause	1 (0.0)	1 (0.1%)	2 (0.1)	2 (0.2%)	1.19 (1.18; 1.21), < 0.001
**Hospitalization**	**230 (10.7)**	140 (17.4%)	**453 (19.0)**	222 (24.9%)	0.85 (0.72; 1.01), 0.073
Hospitalization cardiovascular event	135 (6.3)	81 (10.1%)	295 (12.4)	158 (17.7%)	0.83 (0.70; 1.00), 0.045
Hospitalization other event	80 (3.7)	56 (7.0%)	102 (4.3)	72 (8.1%)	0.98 (0.81; 1.19), 0.854
Hospitalization unknown cause	1 (0.0)	1(0.1%)	0 (0.0)	0(0.0%)	-
**Rehabilitation clinic stay**	**17 (0.8)**	16 (2.0%)	**23 (1.0)**	23 (2.6%)	1.07 (0.98; 1.16), 0.137
Rehabilitation clinic stay due to cardiovascular event	8 (0.4)	7 (0.9%)	13 (0.5)	13 (1.5%)	1.19 (0.98; 1.44), 0.084
Rehabilitation clinic stay other reason	9 (0.4)	9 (1.1%)	10 (0.4)	10 (1.1%)	1.01 (0.95; 1.07), 0.836

*RR* relative risk estimated by poisson regression weighted by a propensity score in order to compare event rates between PCSK9-mAb receiver and PCSK9-mAb non receiver

Characteristics of patients after propensity score matching are shown in Supplementary Table 1, and propensity score–adjusted clinical event rates, both in total and per 100 patient-years, are presented in Supplementary Table 2.

In an ancillary “per protocol” analysis, clinical events were reassigned based on their temporal relationship to treatment. This analysis revealed no major differences compared to the original analysis, confirming the robustness of the findings.

Higher age and comorbidities—such as diabetes mellitus, arterial hypertension, or peripheral arterial disease—were positively associated with the occurrence of cardiovascular events (see Supplementary Table 3).

### Quality of life

On the EuroQoL visual analogue scale (VAS), ranging from 0 (minimum) to 100 (maximum), the quality of life remained stable, with similar mean values between the groups (ranging across visits from 73 to 76 points). The median VAS score was consistently 80.0 in both groups across all time points.

## Discussion

This study highlights four key findings regarding the real-world use of PCSK9-mAb therapy in high-risk dyslipidemia patients: First, treatment persistence was high, with over 90% of patients remaining on PCSK9-mAb therapy since treatment initiation over a 3-year period. Second, PCSK9-mAb therapy led to substantial and sustained reductions in LDL-C levels, superior to non-PCSK9-mAb treatment regimens, with median values consistently below 58 mg/dL throughout the follow-up. Third, PCSK9-mAb recipients experienced fewer hospitalizations due to cardiovascular events compared to non-recipients, underscoring the therapy’s potential clinical benefits. Fourth, LDL-C lowering was improved over time in the non-PCSK9-mAb treatment group.

### PCSK9-mAb treatment allocation and study design considerations

It is important to note that the roughly equal distribution of patients receiving versus not receiving PCSK9-mAb therapy at baseline was a predefined objective of the study design to enable comparative analysis. Therefore, the number of patients in the two groups reflects the planned 50:50 ratio and should not be interpreted as indicative of real-world treatment rates or as evidence of PCSK9-mAb underuse. Nonetheless, existing literature and previous baseline analyses from the PERI-DYS cohort indicate that many patients in both the USA and Europe who meet guideline-based eligibility criteria remain untreated with PCSK9-mAb in routine clinical practice [11, 12]. Contributing factors to this treatment gap—such as restrictive reimbursement policies, patient hesitancy, and complex prescribing requirements—have been discussed previously [11], but were not explored in our study.

### PCSK9-mAb persistence

Early reports based on pharmacy claims in the USA between 2015 and 2019 reported that only 76% of patients were persistent at 6 months after PCSK9-mAb initiation [13]. In a similar analysis of US patients in Medicare and a commercial health plan in 2016, 42.6% of patients discontinued their PCSK9-mAb during the 180 days of follow-up [14]. More recently, observational studies as ours have reported much higher persistence respectively lower discontinuation rates. Our PERI-DYS study, with a follow-up period of 36 months—one of the longest among similar PCSK9-mAb studies—shows a persistence rate of over 90% at the study’s conclusion. Comparatively, the HEYMANS study demonstrated a persistence rate of 92% at 30 months [15]. Similarly, the US-American GOULD study, which included 5006 patients with ASCVD treated with various lipid-lowering therapies, reported a persistence rate of 92% for PCSK9-mAb at 24 months [16]. In line with these findings, the prospective observational ZERBINI study reported a 92% persistence rate with evolocumab after 12 months [17], and the first Italian AT-TARGET-IT registry a persistence of PCSK9-mAb of 97.5% at 18 months [18]. Notably, the observational study design itself likely contributes to the high persistence rates in these studies. Structured monitoring and systematic documentation of drug use, inherent to the study framework, may create a study effect that reinforces treatment adherence beyond what might occur in routine clinical practice [19].

Our analysis identified baseline statin intolerance as a predictor of PCSK9-mAb discontinuation, while other demographic and clinical factors appeared to have limited influence. As potential reason, patients with a history of statin intolerance may have heightened sensitivity or a lower tolerance for perceived side effects of any lipid-lowering therapies, including PCSK9mAb. This heightened awareness or fear of adverse effects could contribute to early discontinuation [20]. Additionally, the complexity of managing statin-intolerant patients might play a role: These individuals often have a more challenging lipid management history, frequently involving multiple prior treatment failures and subsequent treatment attempts with new agents. This history of therapeutic difficulties with statins could lead to frustration, scepticism, or reduced motivation to persist with new treatments, even when they represent innovative approaches like PCSK9-mAbn. Further research is needed to explore these hypotheses and to identify strategies that can improve adherence among this subgroup, such as enhanced patient education, addressing concerns about side effects, and tailored support for managing complex treatment histories.

### Long-term effects on LDL-C

We have previously reported that in our study, PCSK9-mAb receivers were characterized by higher baseline LDL-C and a higher portion of statin intolerance (67.3% versus 15.3%)compared to those qualified for but not-receiving PCSK9-mAb treatment [9]. PCSK9-mAb receivers versus non-receivers were more likely to have CAD, less likely to have PAD, and less likely to have diabetes mellitus. Patients in the PCSK9-mAb groups received fewer concomitant statins, which is in line with the far higher portion of statin-intolerant patients in this group. Further, in PCSK9-mAb receivers, on-treatment LDL-C levels were continuously lower during the study [9]. Given these differences, the raw LDL-C values are misleading for group comparisons. The propensity score adjustment of LDL-C values using logistic regression based on baseline demographic and clinical characteristics, such as comorbidities and additional treatments, revealed that in the PCSK9-mAb receiver group, the LDL-C values at all time points were substantially lower compared to the non-receiver group. In PERI-DYS, propensity-adjusted LDL-C values under PCSK9-mAb therapy were reduced substantially from baseline and remained consistently low during the complete follow-up, in the range of 52–58 mg/dL. They were consistently lower compared to the non-receiver group who had LDL-C values during follow-up in the range of 85–68 mg/dL, showing a continuous trend towards lower values over subsequent visits. For comparison, in the evolocumab cardiovascular outcomes trial (FOURIER) study who had a similar 3-year follow-up period and a strict statin management, patients on evolocumab had an LDL-C in the range of 35mg/dL [4]. Notably, in the PERI-DYS cohort, statin comedication within the PCSK9-mAb group was lower and baseline LDL-C levels were significantly higher. Other observational studies in Germany, such as a monocenter study from the Charité (up to 68 weeks follow-up) [21], PEARL (24 weeks) [22], and HYDRA-FH (52 weeks) [23], have shorter follow-up periods than our registry, or long-term data have not yet been published (e.g., CaRe high registry up to 3 years) [24].

### Clinical events

Cardiovascular events are frequent in high-risk cardiovascular patients. For example, following the index procedure, approximately 20% of patients had at least one subsequent ASCVD-related procedure in the first 12 months, and approximately 50% had an ASCVD-related re-hospitalization [25]. In our study, there were fewer hospitalizations due to CV events in the receiver group and a non-significant numerical trend towards fewer major adverse cardiovascular and cerebrovascular events (MACCE), likely related to lower LDL-C over time and/or different distribution of factors associated with CV events at baseline. However, it is important to note that the observed differences in clinical event rates may underestimate the true benefit of PCSK9-mAb treatment. Patients receiving PCSK9-mAb were more likely to have a higher baseline CV risk, as indicated by higher LDL-C levels, a greater prevalence of statin intolerance, and a higher frequency of prior CV events, which may have led to preferential prescription of PCSK9-mAb. Consequently, despite this initial risk imbalance, the observed reduction in cardiovascular hospitalizations suggests a meaningful clinical benefit of PCSK9-mAb therapy.

In the randomized controlled FOURIER trial, the primary endpoint primary composite end point of CV death, myocardial infarction, stroke, hospitalization for unstable angina, or coronary revascularization over a median follow-up of 26 months (maximum 3.2 years) occurred in 9.8% of evolocumab patients compared to 11.3% in the placebo/standard of care group, with most patients receiving potent statins [4].

Observational studies have reported varying clinical event rates, such as the Italian AT-TARGET-IT registry, where after a median observation period of 19.3 months, 2.4% of patients on different PCSK9i experienced ACS, 2.3% were hospitalized, 0.4% had a stroke, and 0.8% received a new diagnosis of heart failure [18]. Notably, several large studies including the European SANTORINI [26] or HEYMANS studies [27], the US GOULD study [16], or the Canadian ODYSSEY APPRISE study [28] have not yet reported clinical event outcomes, making it unclear whether the observed LDL-C reductions translate into improved cardiovascular outcomes.

### Quality of life

Quality of life (QoL), assessed using the established EuroQoL (EQ) visual analogue scale (VAS), was relatively high in our study cohort, ranging from 73 to 76 points, despite 69% of PCSK9-mAB receivers and 18.0% of non-receivers reported statin intolerance. For comparison, recent data from the German Statin Intolerance Registry (SIR), which included patients with a mean age of 66 years and 58% women, reported a significantly lower mean EQ VAS score of 65 (SD 18) [29]. Furthermore, the general German population, with a younger mean age of 47 years and a balanced gender distribution (48% males), demonstrates a mean EQ VAS score of 85 points [30]. Several factors may explain the relatively high QoL scores observed in our cohort. One possibility is a “study effect,” where structured monitoring and increased attention from healthcare professionals enhanced patient engagement and adherence to treatment plans, thereby improving perceived health status. Additionally, selection bias may have played a role; patients included in the SIR, for example, may have been more likely to attend specialized clinics due to manifest issues such as muscle pain or other symptoms negatively impacting their QoL.

### LDL-C lowering in the non-PCSK9-mAb treatment group

Patients in the non-PCSK9-mAb treatment group exhibited improved LDL-C lowering over time as well. Median adjusted LDL-C levels in the non-PCSK9-mAb group continuously decreased from 106.0 mg/dL at baseline to 68.4 mg/dL at 3 years. However, the recommended treatment goals were not reached by the majority of the very high-risk patients in that group.

### Methodological considerations

The PERI-DYS study was conducted within the framework of German G-BA eligibility criteria for reimbursement of PCSK9-mAb-therapy as defined by the G-BA, which define standard prescription guidelines for PCSK9-mAb. Given that similar reimbursement restrictions exist in many other countries, our findings may have broader applicability beyond Germany [31].

Nevertheless, as an observational study, PERI-DYS is subject to inherent limitations, particularly the lack of randomization [32]. This introduces the potential for selection bias, as physicians may preferentially prescribe PCSK9-mAb to patients with a higher perceived cardiovascular risk, which could lead to an underestimation of treatment benefit when comparing clinical events. The observed reductions in cardiovascular hospitalizations and trends in MACCE rates should therefore be interpreted in the context of this baseline risk disparity.

To mitigate confounding effects, a propensity score adjustment was applied when evaluating the LDL-C course. This statistical approach accounted for differences in baseline characteristics, such as comorbidities and additional lipid-lowering treatments, allowing a more balanced comparison between groups. However, clinical events and adverse events were not adjudicated, and treatment intensification over time within the non-PCSK9-mAb group may have influenced event rates.

The hazard ratio for discontinuation (HR 2.3, 95% CI 1.2–4.4) should be interpreted with caution due to the low number of events (*n* = 68).

Despite these limitations, the study provides valuable real-world insights into long-term LDL-C control, treatment persistence, and clinical outcomes in high-risk dyslipidemia patients receiving PCSK9-mAb therapy.

A key factor to consider is that, in the early phase of this study, there was a higher proportion of patients with familial hypercholesterolemia and complete statin intolerance. This was the primary patient group for PCSK9-mAbs before the FOURIER study outcome label became widely established in clinical practice.

Despite these limitations, the study provides valuable real-world insights into long-term LDL-C control, treatment persistence, and clinical outcomes in high-risk dyslipidemia patients receiving PCSK9-mAb therapy.

In conclusion, the study demonstrates the real-world efficacy of PCSK9-mAb therapy in achieving sustained LDL-C reduction and reducing cardiovascular hospitalization rates, highlighting its role in managing high-risk dyslipidemia patients. Notably, the therapy enables patients with very high baseline LDL-C levels to move significantly closer to target values. Additionally, the high persistence rates observed in this study reinforce its practicality and therapeutic value in real-world clinical settings.

## Supplementary Information

Below is the link to the electronic supplementary material.

Supplementary File 1 (DOCX 769 KB)

## Data Availability

Qualified researchers may request data from Amgen clinical studies. Complete data are available at the following: https://wwwext.amgen.com/science/clinical-trials/clinical-data-transparency-practices/.

## Appendix

**Table:**

Title	Name	Surname	Affiliation	Postal Code	City
Dr. med.	Al-Zoebi	Ayham	Internistische Praxis	04779	Wermsdorf
Dr. med	Amann	Berthold	Franziskus-Krankenhaus BerlinAkademisches Lehrkrankenhaus der CharitéUniversitätsmedizin BerlinStudienabteilung der Klinik für Innere Medizin, Angiologie, Diabetologie	10787	Berlin
Dr. med.	Axthelm	Christoph	Cardiologicum Dresden/PirnaHausärztlich-Kardiologisches MVZ"Am Felsenkeller"GmbH	01796	Pirna
Priv.-Doz. Dr. med.	Bäumer	Anselm	Praxis Dres. Bäumer/Dodos/Klaer	51065	Köln
Dr.med.	Beckermann	Johannes	Marienhospital Vechta gGmbH	49377	Vechta
Dr. med.	Beigel	Andrea	Eickenhof Dialyse/Innere Medizin/Nephrologie	30851	Langenhagen
Prof. Dr. med.	Birkenfeld	Andreas	Medizinische Fakultät der Technischen Universität Dresden Med. Klinik und Poliklinik IIIz. Hd. Frau Schrapel	01307	Dresden
Dr. med.	Bischoff	Steffen	Nephrologische Gemeinschaftspraxis in Dresden-Johannstadt Dr. Bischoff/Dr. Müller	01307	Dresden
Dr. med.	Borucki	Katrin	Universitätsklinikum Magdeburg Institut für klinische Chemie und Pathobiochemie/Lipidambulanz	39120	Magdeburg
Dr. med.	Brandt	Michael	OGD MVZ Neuruppin II	16816	Neuruppin
Dr. med.	Brode	Markus	Kardiologische Praxis	08060	Zwickau
Prof. Dr. med.	c/o Dr. Jessica Halfen	Dirk Raddatz	Universitätsmedizin Göttingen Studienzentrum UMG	37075	Göttingen
Dr. med.	Eberhard	Karin	Cardiologicum Dresden/Pirna	01277	Dresden
Dr. med.	Ebert	Hans-Holger	Herz Riesa, Gemeinschaftspraxis Dres. Stenzel, Ebert & Otto	01587	Riesa
Dr. med.	Fendler	Franz-Rudolf	Integriertes Diabetes-Zentrum Hannover Diabetes	30159	Hannover
Dr. med.	Gebauer	Katrin	Universitätsklinikum Münster Abteilung für Angiologie	48149	Münster
PD Dr. med.	Gerth	Jens	Heinrich-Braun-Klinikum gemeinnützige GmbH Klinik für Innere Medizin II	08060	Zwickau
Dr. med.	Gysan	Detlef	Dres. Heinzler/Gysan/May	51105	Köln
Dr. med.	Haas	Johannes	CIMS Studienzentrum Bamberg GmbH Drs. Wagner/Kirschner/Haas Innere Medizin/Kardiologie	96049	Bamberg
Prof. Dr. med.	Hanefeld	Markolf	EPIDAURUS | Praxis für Diagnostik, Vorsorge & Therapie	01067	Dresden
Dr. med.	Hohenstein	Bernd	Nephrologisches Zentrum Villingen-Schwenningen	78052	Villingen-Schwenningen
Dr. med.	Jahnke	Norbert	Herzklinik Ulm	89077	Ulm
Dr. med.	Jansen	Armin	Praxis fürKardiologie und Angiologie	42117	Wuppertal
Priv.-Doz. Dr. med.	Janssen	Ulf	Kliniken Maria Hilf Krankenhaus St. Franziskus	41063	Mönchengladbach
Dr. med.	Keßler	Sebastian	iKardio Kardiologische Praxis PD Dr. med. Holger Diedrichs und Sebastian Keßler	50354	Hürth
Dr. med.	Killat	Holger	Kardiologische Praxis	67457	Haßloch
Dr. med.	Koschker	Ann-Cathrin	Uniklinik Würzburg Endokrinologie	97080	Würzburg
Dr. med.	Laufs	Ulrich	Universitätsklinikum Leipzig Klinik für Kardiologie und Angiologie	04103	Leipzig
Prof. Dr. med.	Mann	Alexander	Endokrinologikum Frankfurt am Main	60596	Frankfurt
Dr. med.	Meesters	Rüdiger	GemeinschaftspraxisDr. Rüdiger Meesters, Internist-Sportmedizin Dr. Gudrun Mendler - Angiologin	84137	Vilsbiburg
Dr. med.	Menzel	Frank	Praxis Menzel	06847	Dessau-Roßlau
Dr. med.	Meyer	Sven	Forschungs- und Fortbildungsinstitute OM GmbH Kardiologe	49377	Vechta
Dr. med.	Nagel	Mato	Zentrum für Nephrologie und Stoffwechsel, Gemeinschaftspraxis	02943	Weißwasser
Dr. med.	Neumann	Volker	Poliklinik Rüdersdorf	15562	Rüdersdorf
Dr. med.	Noé	Sebastian	MVZ München am Goetheplatz in Trägerschaft der med4muc GmbH	80337	München
Dr. med.	Oettler	Wolfram	Angiologie/Hämostaseologie	02827	Görlitz
	Payk	Dietmar	Studien und Fortbildungs - GbR	58455	Witten
Dr.med.	Ries	Wolfgang	Diakonissenkrankenhaus Flensburg, Medizinische Klinik, Innere Medizin, Facharzt für Innere Medizin, Nephrologie und AngiologieÄrztliche Leitung Nephrologie	24939	Flensburg
Dr. med.	Ringel	Jens	DIAMEDIKUM Potsdam	14473	Potsdam
Prof. Dr. med.	Ruef	Johannes	Kardiocentrum Frankfurt an der Klinik Rotes Kreuz	60316	Frankfurt a.M.
Dr. med.	Salbach	Peter	ze:ro PRAXEN GbR	68165	Mannheim
PD Dr. med.	Schettler	Volker	Praxis	37075	Göttingen
Prof. Dr. med.	Schlitt	Axel	Paracelsus-Harz-Klinik Abteilung 1 - Kardiologie, Diabetologie	06485	Quedlinburg, OT-Bad Suderode
Dr. med.	Schön	Norbert	Kardiologie Mühldorf	84453	Mühldorf/Inn
Dr. med.	Schulze	Beate	Praxis	01558	Großenhain
Dr. med.	Schwittay	Andreas	Praxis	4564	Böhlen
Dr. med.	Simon-Wagner	Ilka	Praxis Dr. med. Ilka Simon-Wagner	96215	Lichtenfels
Dr. med.	Sinning	David	Charité - Universitätsmedizin Berlin, Campus Benjamin Franklin Medizinische Klinik für Kardiologie/Lipidambulanz	12203	Berlin
Dr. med.	Spengler	Ulrike	Praxis	04451	Borsdorf OT Panitzsch
Dr. med.	Spens	Antje	MVZ Stoffwechselmedizin Leipzig, Praxis für Endokrinologie, Schwerpunktpraxis Diabetes mellitus	04317	Leipzig
Dr. med.	Spitthöver	Ralf	Dialyse- und Lipidzentrum Nordrhein	45127	Essen
Dr. med	Stach-Jablonski	Ksenija	Klinikum Mannheim GmbH	68167	Mannheim
Dr. med.	Stadelmann	Alexander	Kardiologie am weißen Turm	90402	Nürnberg
Prof. Dr. med.	Steinhagen-Thiessen	Elisabeth	Charité - Universitätsmedizin Berlin,Campus Virchow-Klinikum Charité Centrum Innere Medizin mit Gastroenterologie und Nephrologie CC 13 Lipidambulanz	13353	Berlin
Dr. med.	Stöckl	Alexander	Gemeinschaftspraxis Diedorf	86420	Diedorf
Dr. med.	Taggeselle	Jens	Praxis	04461	Markkleeberg
MD	Tekiyeh	Mohsen	Medizinisches Versorgungszentrum	45136	Essen
Dr. med.	Twisselmann	Thomas	Kardiologie am Tibarg	22459	Hamburg
Dr. med.	Unger	Tilman	MVZ Quedlinburg	38820	Halberstadt
Dr. med.	Weißbrodt	Matthias	Kardiologische Gemeinschaftspraxis, Innere Medizin | Kardiologie	04103	Leipzig
Dr. med	Wild	Beate	Kardiologische Gemeinschaftspraxis am Park Sanssouci	14471	Potsdam
Dr. med.	Wilke	Andreas	Kardiologische Praxis Papenburg	26871	Papenburg
Dr. med.	Wittig	Ina	Praxis für innere Medizin AngiologieGerinnungsstörungen in Leipzig	04109	Leipzig
Prof. Dr. med.	Zugck	Christian	Kardiologische Praxis im Steiner Thor	94315	Straubing
