# Supplementary material for: Treatment persistence, lipid lowering, and 3-year clinical outcomes in patients at very high cardiovascular risk on PCSK9 monoclonal antibodies
Source: Clin Res Cardiol. 2025 Aug 4;115(2):288–303. doi: 10.1007/s00392-025-02719-z (PMC12823742; doi:10.1007/s00392-025-02719-z)
Supplement: Supplementary file 1 — (DOCX 769 KB) [file 392_2025_2719_MOESM1_ESM.docx]

**Supplementary Materials**

**Parhofer et al.
TREATMENT PERSISTENCE, LIPID LOWERING, AND 3-YEAR CLINICAL OUTCOMES IN PATIENTS AT VERY HIGH CARDIOVASCULAR RISK ON PCSK9 MONOCLONAL ANTIBODIES**

**Supplementary Table 1. Characteristics, comorbidities and lipid values at baseline, unweighted and propensity score (PS) weighted**

| **Parameter** | **PCSK9-mAb  receiver** | | **PCSK9-mAb  non-receiver** | | **p value*** |
| --- | --- | --- | --- | --- | --- |
|  | **(n=804)** | | **(n=891)** | |  |
|  | n(%)  unweighted | % Propensity score weighted | n(%)  unweighted | % Propensity score weighted |  |
| Homozygous familial hypercholesterolemia | 4 (0.5%) | 0.4% | 1 (0.1%) | 0.1% | 0.94 |
| Heterozygous familial hypercholesterolemia | 54 (6.7%) | 7.7% | 41 (4.6%) | 8.0% |  |
| Non-familial/mixed dyslipidemia | 746 (92.8%) | 91.8% | 849 (95.3%) | 91.9% |  |
| Sex, female | 306 (38.1%) | 34.7% | 281 (31.5%) | 33.7% | 0.75 |
| Age (years, mean, SD) | 62.3 (10.3) | 62.2 (10.2) | 64.3 (12.3) | 62.5 (10.2) | 0.69 |
| BMI, kg/m^2^ | 28.4 (4.7) | 28.5 (4.7) | 28.2 (4.7) | 28.2 (4.7) | 0.25 |
| **Comorbidities, %** |  |  |  |  |  |
| Arterial hypertension | 637 (80.0%) | 82.4% | 748 (84.7%) | 83.9% | 0.49 |
| Diabetes mellitus | 194 (24.4%) | 26.7% | 256 (28.7%) | 26.2% | 0.87 |
| CAD | 594 (74.6%) | 72.6% | 608 (68.9%) | 71.7% | 0.76 |
| CVD | 108 (13.6%) | 12.1% | 96 (10.9%) | 13.5% | 0.56 |
| PAD | 102 (13.0%) | 14.7% | 198 (22.9%) | 16.7% | 0.38 |
| Any combination CAD, CVD, PAD | 659 (82.0%) | 81.8% | 721 (80.9%) | 82.0% | 0.93 |
| Chronic kidney disease | 86 (10.8%) | 9.8% | 104 (12.0%) | 12.0% | 0.36 |
| * Test of propensity score weighted data | | | | | |

**Supplementary Table 2. Propensity score adjusted clinical events in total and rate by 100 patient years**

| **Event** | **PCSK9-mAb  receiver** | | **PCSK9-mAb  non-receiver** | |
| --- | --- | --- | --- | --- |
|  | **rate per 100 years** | **% Patients with event** | **rate per 100 years** | **% Patients with event** |
| **Any event** | 15.0 | 21.9 | 19.5 | 23.1 |
| **Any cardiovascular event** | 9.7 | 13.2 | 13.2 | 15.6 |
| **Severe cardiac and cerebrovascular  complication (MACCE)** | 2.8 | 5.6 | 3.2 | 6.2 |
| **Death** | 0.7 | 1.9 | 0.8 | 2.0 |
| Death from cardiovascular cause | 0.1 | 0.3 | 0.2 | 0.5 |
| Death from non-cardiovascular cause | 0.1 | 0.3 | 0.2 | 0.5 |
| Death from unknown cause | 0.5 | 1.3 | 0.4 | 1.0 |
| **Acute coronary syndrome** | 2.2 | 5.3 | 2.6 | 4.7 |
| Acute coronary syndrome STEMI | 0.2 | 0.5 | 0.5 | 1.1 |
| Acute coronary syndrome NSTE-ACS | 0.2 | 0.6 | 0.8 | 1.4 |
| Acute coronary syndrome angina pectoris | 1.7 | 4.3 | 1.1 | 2.8 |
| Acute coronary syndrome unknown | 0.1 | 0.1 | 0.1 | 0.3 |
| **Cerebrovascular event** | 0.8 | 1.4 | 1.2 | 1.9 |
| Cerebrovascular event ischemic stroke | 0.1 | 0.3 | 0.1 | 0.2 |
| Cerebrovascular event hemorrhagic stroke | 0.0 | 0.0 | 0.0 | 0.0 |
| Cerebrovascular event TIA | 0.1 | 0.3 | 0.0 | 0.0 |
| Cerebrovascular event unknown cause | 0.1 | 0.3 | 0.0 | 0.0 |
| Cerebrovascular event other cause | 0.1 | 0.1 | 0.4 | 1.0 |
| **Hospitalization** | 10.8 | 18.8 | 14.9 | 20.8 |
| Hospitalization cardiovascular event | 6.7 | 11.3 | 9.5 | 12.9 |
| Hospitalization other event | 4.3 | 8.6 | 4.2 | 8.0 |
| Hospitalization unknown cause | 0.0 | 0.1 | 0.0 | 0.0 |
| **Rehabilitation clinic stay** | 0.8 | 2.0 | 0.8 | 2.0 |
| Rehabilitation clinic stay due to cardiovascular event | 0.3 | 0.7 | 0.5 | 1.2 |
| Rehabilitation clinic stay other reason | 0.5 | 1.3 | 0.3 | 0.8 |

**Supplementary Table 3. Association of age, female sex and comorbidities with outcomes (univariable analysis)**

|  | **Any cardiovascular events** | | | **Severe cardiac and cerebrovascular complication (MACCE)** | | | **Cardiovascular hospitalizations** | | |
| --- | --- | --- | --- | --- | --- | --- | --- | --- | --- |
|  | **HR** | **95%CI** | **p value** | **HR** | **95%CI** | **p value** | **HR** | **95%CI** | **p value** |
|  |  |  |  |  |  |  |  |  |  |
| Age in years | 1.03 | 1.01 ; 1.04 | <0.001 | 1.02 | 1.00 ; 1.04 | 0.023 | 1.03 | 1.01 ; 1.04 | <0.001 |
| Female gender | 0.78 | 0.60 ; 1.02 | 0.070 | 0.58 | 0.38 ; 0.88 | 0.011 | 0.82 | 0.63 ; 1.09 | 0.172 |
| BMI | 0.99 | 0.96 ; 1.02 | 0.433 | 1.03 | 0.99 ; 1.06 | 0.182 | 1.00 | 0.97 ; 1.03 | 0.866 |
|  |  |  |  |  |  |  |  |  |  |
| Diabetes mellitus | 1.40 | 1.11 ; 1.77 | 0.005 | 1.82 | 1.33 ; 2.50 | <0.001 | 1.39 | 1.08 ; 1.78 | 0.010 |
| Artrial hypertension | 1.39 | 1.02 ; 1.91 | 0.038 | 1.30 | 0.83 ; 2.04 | 0.251 | 1.44 | 1.02 ; 2.01 | 0.036 |
| Heart failure | 1.30 | 1.02 ; 1.65 | 0.033 | 1.85 | 1.37 ; 2.48 | <0.001 | 1.19 | 0.91 ; 1.56 | 0.205 |
| Respiratory insufficiency | 1.15 | 0.86 ; 1.53 | 0.354 | 1.39 | 0.99 ; 1.95 | 0.055 | 1.08 | 0.79 ; 1.46 | 0.632 |
| Coronary artery disease | 1.36 | 1.04 ; 1.79 | 0.023 | 2.19 | 1.44 ; 3.33 | <0.001 | 1.25 | 0.94 ; 1.66 | 0.128 |
| Cerebrovascular disease | 0.96 | 0.67 ; 1.38 | 0.825 | 1.05 | 0.65 ; 1.69 | 0.856 | 0.91 | 0.62 ; 1.35 | 0.649 |
| Peripheral arterial disease | 2.14 | 1.74 ; 2.64 | <0.001 | 1.81 | 1.33 ; 2.45 | <0.001 | 2.30 | 1.85 ; 2.86 | <0.001 |
| Atrial fibrillation/flutter | 1.66 | 1.23 ; 2.23 | 0.001 | 1.32 | 0.84 ; 2.06 | 0.226 | 1.75 | 1.28 ; 2.38 | <0.001 |
| Renal failure | 1.34 | 1.02 ; 1.76 | 0.038 | 1.50 | 1.06 ; 2.12 | 0.021 | 1.32 | 0.99 ; 1.76 | 0.055 |

**Supplementary material (methods): Propensity score estimation**

Propensity score (PS) methods aim to mimic certain attributes of randomized designs from observational data. In a valid PS design where all baseline confounders are measured, the confounders would be balanced, allowing the treatment status to be considered as if it were randomly assigned. [Suppl Ref 1] To evaluate the LDL-C course, in this analysis a PS was estimated to balance clinical characteristics between the two treatment groups using logistic regression. [Supple Ref 2] The PS included baseline demographic and clinical characteristics, including comorbidities and additional treatments, to account for potential confounding factors. An inverse probability weight was calculated for the two treatment groups: for patients receiving PCSK9-mAb therapy, at baseline (pre-treated and newly treated) the weight was defined as 1/PS, and for those not receiving PCSK9-mAb therapy, the weight was 1/(1-PS). This weighting approach aimed to address the non-randomized allocation of treatments and ensure a balanced comparison between the groups.

The following parameters were included in the logistic regression model in order to estimate the propensity score: Diagnostic categories, female sex, age, BMI, comorbidities (arterial hypertension, diabetes mellitus, CAD, CVD, PAD, chronic kidney disease) and statin use, ezetimibe use and statin intolerance

1. This model discriminated well between PCSK9-mAb receiver and PCSK9-mAb non-receiver (Area under Curve of 0.83). The model was also calibrated (chi2=1251, p=0.509).
2. A considerable overlap in the PS-score distribution between PCSK9-mAb receiver and PCSK9-mAb non-receiver exists.


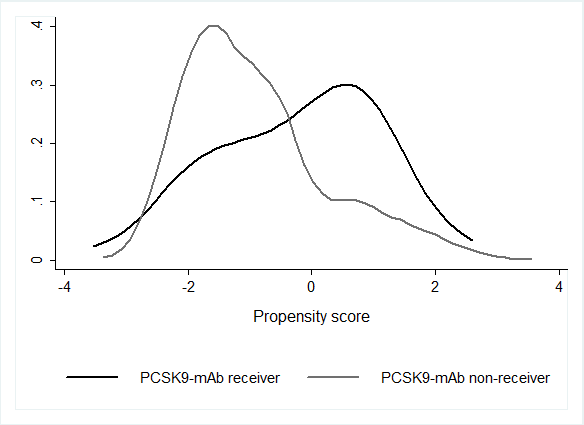


1. The propensity score balanced the baseline distribution between PCSK9-mAb receiver and PCSK9-mAb non-receiver, as shown by the standardized mean for continuously distributed variables and difference in proportion for categorical variables.

|  | unweighted | Weighted by propensity score |
| --- | --- | --- |
|  | Standardized mean difference  / Difference in proportion in % | |
| **Demographics and indication** |  |  |
| Homozygous familial hypercholesterolemia | -0.39 | -0.40 |
| Heterozygous familial hypercholesterolemia | -2.11 | -0.06 |
| Non-familial/mixed dyslipidemia | 2.50 | 0.46 |
| Sex, female | -6.52 | -0.51 |
| Age (years, mean, SD) | 0.86 | 0.19 |
| BMI, kg/m^2^ | -0.15 | -0.24 |
|  |  |  |
| **Comorbidities, %** |  |  |
| Arterial hypertension | 4.69 | 1.26 |
| Diabetes mellitus | 6.89 | -0.14 |
| CAD | -5.77 | -1.13 |
| CVD | -2.68 | 1.15 |
| PAD | 9.63 | 2.25 |
| Chronic kidney disease | 1.16 | 2.07 |
|  |  |  |
| **Medications** |  |  |
| Statin use | -7.9 | -3.5 |
| Ezetimibe use | 35.7 | 15.8 |

**Supplementary references**:

Suppl Ref 1. Wan F. Propensity Score Matching: should we use it in designing observational studies? BMC Med Res Methodol. 2025;25:25.

Suppl Ref 1. Austin PC (2014) A comparison of 12 algorithms for matching on the propensity score. Stat Med 33:1057–1069
